# The Contribution of Neighbourhood Material and Social Deprivation to Survival: A 22-Year Follow-up of More than 500,000 Canadians

**DOI:** 10.3390/ijerph10041378

**Published:** 2013-04-02

**Authors:** Nancy A. Ross, Lisa N. Oliver, Paul J. Villeneuve

**Affiliations:** 1 Department of Geography, McGill University, 805 Sherbrooke St. West, Montreal, QC H3A 2K6, Canada; 2 Health Analysis Division, Statistics Canada, Ottawa, ON K1A 0T6, Canada; E-Mail: Lisa.Oliver@statcan.ca; 3 Population Studies Division, Health Canada, Ottawa, ON K1A 0K9, Canada; E-Mail: paul.villeneuve@hc-sc.gc.ca; 4 Division of Occupational and Environmental Health, Dalla Lana School of Public Health, University of Toronto, Toronto, ON M5T 3M7, Canada

**Keywords:** neighbourhood deprivation, mortality, survival analysis, immigrant neighbourhoods, Canada

## Abstract

*Background*: We examined the incremental influence on survival of neighbourhood material and social deprivation while accounting for individual level socioeconomic status in a large population-based cohort of Canadians. *Methods*: More than 500,000 adults were followed for 22 years between 1982 and 2004. Tax records provided information on sex, income, marital status and postal code while a linkage was used to determine vital status. Cox models were used to estimate hazard ratios (HR) for quintiles of neighbourhood material and social deprivation. *Results*: There were 180,000 deaths over the follow-up period. In unadjusted analyses, those living in the most materially deprived neighbourhoods had elevated risks of mortality (HR_males_ 1.37, 95% CI: 1.33–1.41; HR_females_ 1.20, 95% CI: 1.16–1.24) when compared with those living in the least deprived neighbourhoods. Mortality risk was also elevated for those living in socially deprived neighbourhoods (HR_males_ 1.15, CI: 1.12–1.18; HR_females_ 1.15, CI: 1.12–1.19). Mortality risk associated with material deprivation remained elevated in models that adjusted for individual factors (HR_males_ 1.20, CI: 1.17–1.24; HR_females_ 1.16, CI: 1.13–1.20) and this was also the case for social deprivation (HR_males_ 1.12, CI: 1.09–1.15; HR_females_ 1.09, CI: 1.05–1.12). Immigrant neighbourhoods were protective of mortality risk for both sexes. Being poor and living in the most socially advantageous neighbourhoods translated into a survival gap of 10% over those in the most socially deprived neighbourhoods. The gap for material neighbourhood deprivation was 7%. *Conclusions*: Living in socially and materially deprived Canadian neighbourhoods was associated with elevated mortality risk while we noted a “healthy immigrant neighbourhood effect”. For those with low family incomes, living in socially and materially deprived areas negatively affected survival beyond their individual circumstances.

What is already known on this subject?

- Canadians enjoy a high standard of living and enviable life expectancy but there are persistent social and economic health disparities that may be associated with neighbourhood of residence.
- The international scientific knowledge base of neighbourhood influences on human health has matured but few studies have examined population-based cohorts with sufficient follow-up periods.

What this study adds?

- Neighbourhood material and social deprivation is associated with long term mortality risk in the Canadian population.
- Canadian immigrant neighbourhoods are protective of the health of both immigrants and non-immigrants suggesting the presence of a “healthy immigrant neighbourhood effect”.
- Income inequality and income segregation increased over the study period suggesting that Canadian neighbourhoods, while historically protective of the health of their populations (especially when compared with American neighbourhoods), may become increasingly important factors in the creation of health disparities in Canada.

## 1. Introduction

Neighbourhoods have been hypothesized to influence health status via many pathways. The major way of thinking of neighbourhood effects has been to understand the neighbourhood as a site for “multiple jeopardy” or “deprivation amplification” [1]. This theory is based on the premise that individual poverty is compounded by the attributes of the poor neighbourhood, which might include both material and social characteristics: underinvestment in neighbourhood services and public goods; exposure to noise and pollutants, crime, conflict or disarray; socialization effects on behaviour and transmission of health-compromising social norms; social isolation and isolation from economic opportunity.

Early prospective research on the relationship between both material and social deprivation and mortality comes from the Alameda County Study. A paper published in 1987 [2] showed mortality risk to be significantly elevated for Oakland, CA residents living in federally designated poverty areas compared to those in more affluent neighbourhoods, with a suite of individual level variables (individual SES, health behaviours) unable to budge the areal-level mortality risks. One of the earliest attempts to examine the role of the social environment on mortality was the linkage of the 1983 wave of the Alameda County Study to mortality records for an 11-year follow-up of about 1,000 Alameda County residents [3]. These authors found strong effects of neighbourhood social environment (operationalized as the prevalence of commercial stores and quality housing) on mortality risk, independent of individual level socioeconomic measures and key behavioural measures (smoking, body mass index, drinking behaviour). During the mid and late 1990s and into the most recent decade there were efforts in the United States [4,5,6], the United Kingdom, [7,8,9,10] and other countries in Europe [11,12] to assess mainly area level material deprivation on all-cause mortality. A study that combined three prospective cohort studies (ARIC-USA; Whitehall II-UK; GLOBE-Netherlands) and three population registry-based studies (Helsinki, Turin, Madrid) found that when individual characteristics (sex, occupation, education) were controlled for, the results of “neighbourhood” level unemployment were attenuated but the difference between the highest and lowest neighbourhood quartile hazard ratio remained significant in all but the Whitehall II study (ARIC HR = 1.21; GLOBE HR = 1.46; Whitehall II HR = 1.19; Helinski HR = 1.41; Turin HR = 1.14; Madrid HR = 1.28). On balance, then, across study designs in different countries (prospective cohorts, population-based mortality linkages) we generally find an influence of neighbourhood-level material conditions (measured in many different ways across studies) on all-cause mortality, above and beyond individual characteristics.

Reviews of the body of work investigating the role of neighbourhoods in the production of human health have called for more longitudinal research to better understand the relationship [13,14,15]. The reality though is that few studies capture both individual and neighbourhood level data for a sufficient enough sample to test the effects of neighbourhood characteristics on long term survival. A recent exception is Major and colleagues’ 2010 analysis of more than 200,000 older (aged 50–71) Americans participating in the NIH-AARP Diet and Health Study [16]. This study assessed the net contribution of neighbourhood deprivation on all-cause and cause-specific mortality for both men and women (33,831 deaths, median time of follow-up of 9.5 years) while controlling for individual level educational attainment as well as a suite of behavioural risk factors, the most important of these being smoking. Living in a deprived neighbourhood was associated with elevated risk of overall mortality with the strongest elevated risks for CVD mortality for men.

Past Canadian research has shown that neighbourhoods exert an incremental influence on the health status of Canadians, [17] although the evidence of an association in Canada between neighbourhood deprivation and subsequent mortality has been inconclusive [18,19,20] with comparatively short follow-up times and smaller, regionally based samples. One exception to this is a recent national scale study showing an influence of neighbourhood conditions on mortality suggesting more study is needed to explore this influence further [21]. Canadians enjoy a high standard of living and some of the longest life expectancies in the world yet there are long-documented, persistent health disparities in Canadian society [22,23] that are linked to social and economic disadvantage.

Our aim here is to assess the influence of neighbourhood social and material deprivation on mortality risk for a large cohort of Canadians living in Canada’s most populous province. While individual behavioural variables are not available in this cohort assembled through data linkage, our study has important strengths not shared by previous work: a long mortality follow-up period (22 years), a wide age range of participants capturing working age mortality (previously demonstrated to be very sensitive to contextual indicators [24]), high quality individual level income data from tax records, an explicit focus on two key neighbourhood dimensions of deprivation (material and social) using validated measures, and very large number of deaths for both men and women in the cohort. We also aim to explicitly test the hypothesis of neighbourhood “deprivation amplification” by comparing survival curves of low income individuals living in neighbourhoods of varying degrees of material and social deprivation.

## 2. Methods

The primary data for this study are from the Ontario Tax Cohort which includes 548,000 people selected between 1982 and 1986 and followed until the end of 2004 (The vast majority of cohort (512,000 individuals) was selected in 1982—there were 36,000 additional cohort members added up until 1986). These individuals were selected from Statistics Canada T1 Family file database which includes all Canadians who completed a tax return, or who received tax benefits for children that resided in the house. This database also includes non-filing spouses, partners and children that were identified from tax filing records. A comparison to Canadian census data suggests that the T1 database includes approximately 95% of the Canadian adult population [25]. From this database, we randomly selected individuals who were between the ages of 35 and 85 at baseline, and lived in one of 10 urban areas in Ontario between 1982 and 1986: Hamilton, Kingston, London, Ottawa, Thunder Bay, St. Catharines, Sarnia, Sudbury, Toronto and Windsor. Together, based on 1981 census data, 67% of the Ontario population, 35 years of age and older lived in these 10 urban areas. Given that the objective for sampling was to represent a range of exposures to both varying social and physical environments, the sampling was not directly proportional to city size. Were this not the case, the sample would have been dominated by Toronto residents. For each cohort member, the tax file provided information on age, sex, personal income, family income, marital status and postal code of residence. Four categories of marital status were created: married (reference category); separated, widowed or divorced; single; and missing. Family income at baseline was extracted from the tax file and reported in dollars.

Personal identifying information at an individual-level basis was linked to the Canadian Mortality Database to ascertain vital status, and underlying cause of death between 1982 through 2004. Registration of deaths is the responsibility of the provinces and territories, and the Canadian Mortality Data Base contains information on all deaths among residents that occur in Canada, and as well as states in the US. Previous studies have demonstrated that less than 5% of all deaths are missed through the probabilistic record linkage method that we used to determine vital status [26,27]. The outcome measure was the relative hazard of death for individuals in the cohort. The survival time was defined as the number of days from the start of the study (1 January 1982) until the day of death. Individuals with no recorded death were assumed to be alive (right censored) at the end of follow-up (31 December 2004). Days were divided by 365.25 to represent person years.

### 2.1. Neighbourhood Variables

Data from the 1986 Census were used to assess neighbourhood characteristics of study participants. We selected census data for 1986 as all cohort members were enrolled by then. Census tracts (n = 1,985) were used as proxies for “neighbourhoods” which has been shown in past studies to be a valid approach [17]. Canadian census tracts are designed to be stable, socially homogeneous urban geographic units with populations that range from 2,500 to 8,000 [28]. Statistics Canada’s Postal Code Conversion File [29] was used to determine the census tract of residence based on individuals’ postal codes. Neighbourhood socio-economic status was assessed by a Canadian index which was developed to measure material and social deprivation [30,31]. The “Pampalon Index” was originally assessed based on factor analysis of six variables (% adults without a high school diploma, employment population ratio, average income, population living alone, population separated, widowed or divorced, and lone parent families). The first three variables are combined into an index of material deprivation while the last three were combined into an index of social deprivation. Because the original index was based on areas in the province of Quebec, we conducted a factor analysis to verify that the same factors emerged for the 10 Ontario cities in the current study. We also found that two factors emerged with Eigenvalues over 1 with factor loadings between .66 and .95. The social and material deprivation indices were divided into quintiles for analysis. Two additional neighbourhood variables that were deemed to be influential for the Canadian mortality experience were also measured: the percent of immigrants and percent of Aboriginal Canadians resident in the census tract.

### 2.2. Statistical Methods

Cox survival models were fit to the data using SAS v.9.1 (SAS Inc, Raleigh, NC, USA). Because respondents were selected from 10 cities, the city of entry was added to the models to control for the potential influence of unmeasured factors influencing mortality that may be unique to each city (results not shown). The Efron [32] method was used to correct for tied survival times as estimates are closer to the exact partial likelihood than the estimates obtained from the Breslow method [33]. We calculated sex-specific models owing to the stark differences in mortality rates between men and women and all models were stratified by age (in years) at baseline. Modelling was conducted incrementally to more fully understand the influence of variable additions on the outcome. Model 1 consisted of only individual level characteristics (age, income, marital status). Model 2 included only the full set of neighbourhood variables while Model 3 included individual and neighbourhood level variables. The −2 Log Likelihood is used to assess the model fit. The proportionality assumption of the Cox models were assessed graphically using −2 Log survivor plots. Inspection of the graphs showed that the assumption of proportionality was satisfied. We produced survival curves for individuals with low income (less than $16K per year) living in the lowest and highest quintile of material and social deprivation. Using the Kaplan-Meier method, the log-rank and Wilcoxon tests were used to determine if the survivor curves were different. Proc lifetest in SAS was used for this analysis.

## 3. Results

The cohort consisted of 548,000 urban residents (51% male, 49% female) in Canada’s most populous province—Ontario (Table 1). The mean age at baseline was 51.9 years with the majority of the respondents married (67%). Average family income at baseline was $36,477 for women and $39,499 for men. There were 180,000 deaths recorded in the cohort (104,000 men and 76,000 women). Of those with no death record, 93% had tax data in the final study year (2004) and were censored at the study end date while 7% were censored at an earlier year. Marital status was available for individuals who filed tax returns. In total 7% were missing marital status and most of these were women.

**Table 1:** Characteristics of Ontario tax cohort.

		Percent (%)	Total
*Total*		100	548,000
	Females	49	271,000
	Males	51	278,000
*Dead*		33	180,000
	Females	28	76,000
	Males	37	104,000
*Censored*		67	369,000
*Average Age (SD)*		51.9 (SD 12.34)	
*Marital Status*			
	Married	67	367,000
	Separated, divorced, widowed	18	101,000
	Single	7	40,000
	Missing	7	40,000
*City of Entry*			
	Toronto	11	58,000
	Hamilton	14	75,000
	Kingston	5	29,000
	London	11	58,000
	Ottawa	14	76,000
	Sarnia	5	27,000
	St. Catharines	10	53,000
	Sudbury	10	52,000
	Thunder Bay	9	51,000
	Windsor	12	69,000

In addition to assigning the 1,985 neighbourhoods into quintiles based on their social and material deprivation scores, we assessed the proportion of the immigrant population (mean 21.77%, SD 9.33%) and the proportion of the Aboriginal population (mean 0.77%, SD 1.36%). 

**Table 2:** Survival of urban Ontario residents (age 35 and older) stratified by age at baseline in years (bold = significance at 0.05)—Males.

		Model 1			Model 2			Model 3		
***Variable***		**HR**	**95% CI**		**HR**	**95% CI**		**HR**	**95% CI**	
***Family Income ($10,000)***		**0.953**	0.951	0.955				**0.96**	0.958	0.962
***Marital Status (vs. Married)***										
	**Sep., wid., div.**	**1.359**	1.335	1.384				**1.331**	1.307	1.355
	**Missing**	**0.852**	0.802	0.906				**0.859**	0.808	0.913
	**Single**	**1.46**	1.427	1.494				**1.406**	1.374	1.44
***Neighbourhood deprivation quintile***										
	**Social 2**				1.006	0.982	1.03	1.02	0.996	1.046
	**Social 3**				1.013	0.991	1.036	**1.03**	1.006	1.054
	**Social 4**				**1.078**	1.053	1.104	**1.079**	1.052	1.106
	**Social 5 (most deprived)**				**1.148**	1.122	1.175	**1.122**	1.094	1.151
	**Material 2**				**1.087**	1.058	1.116	**1.039**	1.011	1.068
	**Material 3**				**1.178**	1.148	1.208	**1.096**	1.066	1.126
	**Material 4**				**1.228**	1.196	1.261	**1.115**	1.082	1.148
	**Material 5 (most deprived)**				**1.37**	1.334	1.406	**1.204**	1.167	1.243
***Aboriginal (%)***					**1.013**	1.008	1.018	**1.007**	1.001	1.012
***Immigrants (%)***					**0.996**	0.996	0.997	**0.996**	0.995	0.997
* −*2 Log Likelihood*		1,654,527			1,648,423			1,644,833		
***Change in* −*2LL from Model 1***					−6104.2			−9694.3		

**Table 3:** Survival of urban Ontario residents (age 35 and older) stratified by age at baseline in years (bold = significance at 0.05)—Females.

		Model 1			Model 2			Model 3		
***Variable***		**HR**	**95% CI**		**HR**	**95% CI**		**HR**	**95% CI**	
***Family Income$(10,000)***		**0.966**	0.963	0.969				**0.97**	0.967	0.973
***Marital Status (vs. Married)***										
	**Sep., wid., div.**	**1.269**	1.246	1.293				**1.251**	1.228	1.275
	**Missing**	**0.84**	0.821	0.86				**0.832**	0.813	0.852
	**Single**	**1.258**	1.223	1.293				**1.243**	1.208	1.279
***Neighbourhood deprivation quintile***										
	**Social 2**				**1.041**	1.01	1.073	**1.036**	1.004	1.069
	**Social 3**				**1.071**	1.042	1.102	**1.05**	1.02	1.081
	**Social 4**				**1.089**	1.058	1.122	**1.045**	1.013	1.078
	**Social 5 (most deprived)**				**1.164**	1.131	1.198	**1.087**	1.054	1.121
	**Material 2**				**1.036**	1.004	1.068	1.015	0.983	1.048
	**Material 3**				**1.074**	1.043	1.107	**1.042**	1.009	1.076
	**Material 4**				**1.116**	1.083	1.151	**1.074**	1.038	1.111
	**Material 5 (most deprived)**				**1.199**	1.163	1.236	**1.151**	1.11	1.193
***Aboriginal (%)***					**1.007**	1.001	1.013	1.004	0.997	1.011
***Immigrants (%)***					**0.998**	0.997	0.999	**0.997**	0.996	0.999
* −*2 Log Likelihood*		1,191,329			1,186,940			1,184,496		
***Change in* −*2LL from Model 1***					−4388.2			−6832.8		

The initial model containing only the individual level baseline variables (Table 2, Table 3—Model 1) shows that increasing family income is associated with a reduced risk of death and is statistically significant for both males and females. Each increase in $10,000 of family income is associated with a HR of 0.951 (95% CI 0.951–0.955) for men and 0.966 (95% CI 0.963–0.969) for women. Compared to being married, being single or separated, widowed or divorced increases the risk of death. This pattern holds for both males and females, however the strength of association is greater for males (1.46 (95% CI 1.43–1.49)) than females (1.26 (95% CI 1.22–1.29)).

Compared to living in a neighbourhood with the least social deprivation, living in the 4th or 5th most deprived neighbourhood significantly increased the risk of death for males and living in the 2nd–5th most deprived significantly increased the risk of death for females (Table 2, Table 3—Model 2). For neighbourhood material deprivation, living in the 2nd–5th most deprived compared to the least deprived significantly increased the risk of death for both males and females. The percent of Aboriginal people living in a neighbourhood was associated with an increased risk of death while the percent of immigrants was associated with a reduced risk of death.

**Figure 1:**
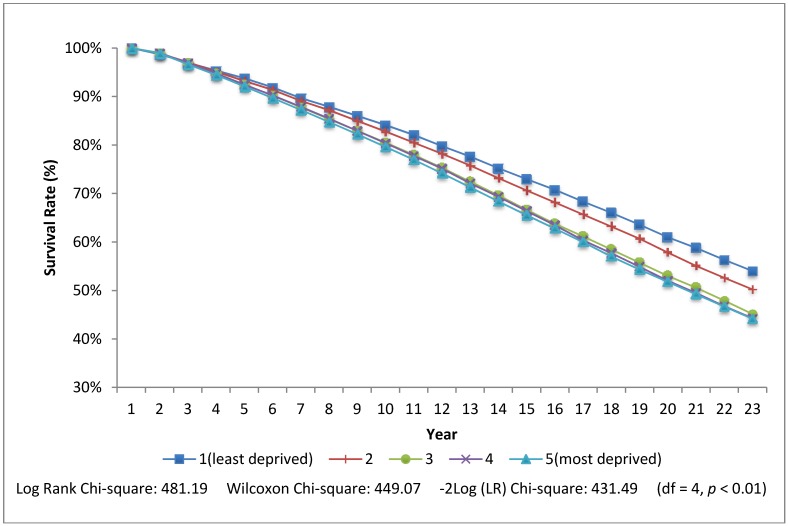
Rate of survival by quintiles of neighbourhood social deprivation for low income individuals.

**Figure 2:**
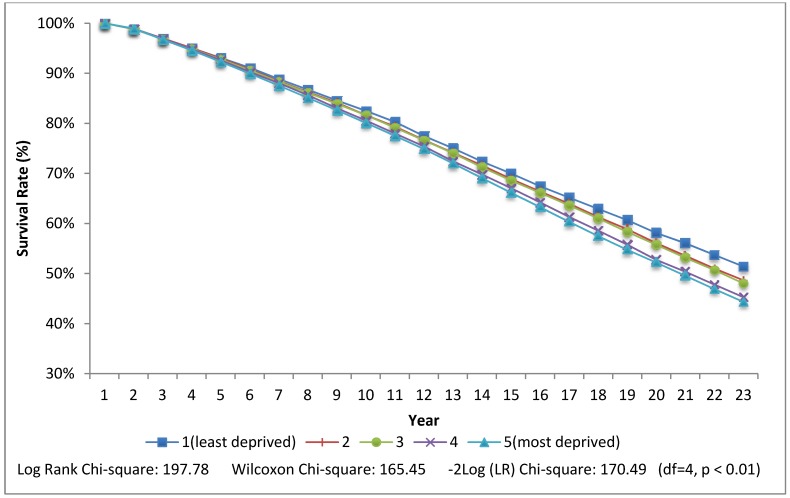
Rate of survival by quintiles of neighborhood material deprivation for low income individuals.

In the final model (Table 2, Table 3—Model 3), relative to living in a neighbourhood that has the least social and material deprivation, living in a more deprived neighbourhood increases the risk of death. These results persist after accounting for individual characteristics (family income, marital status) and city of residence (not shown). A stepwise gradient is evident in which each increasing quintile of deprivation is associated with a higher HR. All are significant except for living in the second least materially deprived quintile for women and the second least socially deprived quintile for men. For both men and women the effect of family income is only slightly attenuated with the addition of the neighbourhood variables. The HR for living in the most socially and materially deprived neighbourhood quintiles is higher for males than females. The HR is 1.20 (95% CI 1.17–1.24) for men in the most materially deprived quintile and 1.15 (95% CI 1.10–1.19) for women. A similar pattern is evident for social deprivation. Survival curves for individuals with a family income less than half of the median ($16K per year) are shown in Figure 1, Figure 2.

For these individuals with low family incomes, 51% of those who lived in the least materially deprived neighbourhoods were alive at the end of follow-up compared to 44% for those living in the most materially deprived neighbourhoods, creating a 7% survival “gap” attributable to material deprivation amplification. The survival gap for poor individuals living in the least socially deprived neighbourhoods (54%) compared to the most socially deprived neighbourhoods (44%) was higher at 10%. The Wilcoxon and log-rank test indicated a significant difference in the survival curves at the *p* = 0.01 level.

## 4. Discussion and Conclusions

Mortality risk in this large population cohort is patterned by both individual level material (family income) and social (marital status) conditions as well as by neighbourhood material and social conditions. We also found evidence of a protective effect on mortality risk of living in an immigrant neighbourhood. This latter finding we suggest may be evidence of a “healthy immigrant neighbourhood effect”—a place-based analog to the well documented healthy immigrant effect that has been shown in Canada and elsewhere [34,35,36,37,38]. The processes by which immigrant neighbourhoods bestow upon their immigrant and non-immigrant residents lower mortality risk are unknown and warrant further investigation.

Our findings are consistent with other large scale efforts to isolate neighbourhood effects on survival, showing an influence of neighbourhood on mortality risk beyond individual level determinants of health. We were also able to demonstrate both material and social neighbourhood deprivation amplification beyond individual level poverty, with social deprivation appearing to exert the slightly stronger effect. These results echo the pioneering work in the role of the social environment on mortality risk in Alameda County, CA [3].

Canadian urban neighbourhoods became increasingly socially and economically homogenous during the study period. Research looking at various time periods back through the mid-1980s found that between-neighbourhood income inequality rose in all Canadian cities, based on total family income (after transfers, before taxes). The increase in between-neighbourhood income inequality was driven largely by a significant rise in neighbourhood earnings inequality. Between 1980 and 1995, real average family neighbourhood earnings fell 20% to 33% (depending upon the city) in poor neighbourhoods, while rising (up to 15%) in the highest income neighbourhoods [39]. This study also showed that employment was increasingly concentrated in high income neighbourhoods and unemployment in low income neighbourhoods and that the effect of social transfers at dampening the neighbourhood inequality gap was only minimal given that social transfers rose in all neighbourhoods during the study period (likely attributable to transfers associated with an aging population). In a Canada-wide study, Ross and colleagues [40] showed an increase in residential segregation by income from 1991 to 1996. Overall, the story of income segregation across Canada during the early to mid-1990s was one of a rise in the spatial separation of income groups across the urban landscape.

There has been little attention to the meaning of social deprivation for the health Canadians to date. This despite the fact that Klinenberg [41] argues that “The incredible rise of living alone is the greatest social change that we’ve failed to name and identify, let alone understand.” Dropping fertility rates, the increasing fluidity of conjugal relationships (divorce, decline of marriage) and population mobility collectively mean that Canadians are increasingly more likely to be alone and our study suggests that social deprivation should be considered as an important determinant of health for Canadians.

A key strength of our study is the large population-based sample that we were able to follow over a long time period. Additional strengths are high quality and highly complete measures of family income as well validated neighbourhood deprivation measures. The trade-off with using large scale administrative data for this type of research is the sacrificing of survey-based measures of health-related behaviours that are related to mortality and socially patterned, chiefly smoking, diet and physical activity. That said, neighbourhoods are modifiable “upstream” factors behind health and mortality disparities and they set the context for the norms and behaviours accepted, as well as the span of income and educational possibilities, the types of health care facilities available, and the types of jobs available to their residents. Future work with the Ontario Tax Cohort will explore the influence of residential mobility on the effect sizes here and can examine the influence of neighbourhood change on mortality. The imperative to continue this avenue of research comes from the notion that Canadian neighbourhoods, while historically protective of the health of their populations (especially when compared with American neighbourhoods [24,42] may become increasingly important factors in the creation of health disparities in Canada.
